# Impact of induction agents and maintenance immunosuppression on torque teno virus loads and year-one complications after kidney transplantation

**DOI:** 10.3389/fimmu.2024.1492611

**Published:** 2024-11-13

**Authors:** Marvin Reineke, Claudius Speer, Christian Bundschuh, Julian A. F. Klein, Lisa Loi, Claudia Sommerer, Martin Zeier, Paul Schnitzler, Christian Morath, Louise Benning

**Affiliations:** ^1^ Department of Nephrology, University Hospital Heidelberg, Heidelberg, Germany; ^2^ Department of Cardiology, Angiology and Pneumology, University Hospital Heidelberg, Heidelberg, Germany; ^3^ Medical Faculty Heidelberg, Department of Infectious Diseases, Virology, Heidelberg University, Heidelberg, Germany; ^4^ German Center for Infection Research, German Center for Infection Research (DZIF), Heidelberg Partner Site, Heidelberg, Germany

**Keywords:** kidney transplantation, immune monitoring, immunosuppression, torque teno virus, immunocompetence

## Abstract

**Background:**

Torque teno virus load (TTVL) is gaining importance as a surrogate parameter to assess immunocompetence in kidney transplant recipients. Although the dynamics of TTVL have been investigated before, the impact of different induction agents and variations in immunosuppressive maintenance therapies on TTVL remain unknown.

**Methods:**

In this retrospective study, TTVL was quantified in 537 plasma or serum samples from 134 patients transplanted between 2018 and 2021. TTVL was examined pre-transplantation and 30-, 90-, 180-, and 360-days post-transplant. To assess the influence of induction therapy on TTVL, 67 patients receiving anti-thymocyte globulin (ATG) induction were matched with 67 patients receiving an interleukin-2 receptor antagonist (IL2-RA) induction in terms of age, sex, and donor modality.

**Results:**

Following transplantation, there was a steep increase in TTVL post-transplant for all patients with peak viral loads at 90 days post-transplant (median TTVL [IQR] 7.97×10^6^, [4.50×10^5^–1.12×10^8^]) followed by subsequently declining viral loads. Compared to patients receiving IL2-RA as induction therapy, patients receiving ATG had significantly higher peak viral loads 3 months post-transplant (median TTVL [IQR] 2.82×10^7^ [3.93×10^6^–1.30×10^8^] vs. median TTVL [IQR] 2.40×10^6^ [5.73×10^4^–2.60×10^7^]; *P*<0.001). Throughout all post-transplant time points, patients receiving additional rituximab for induction along with higher tacrolimus target levels exhibited the highest TTVL.

Patients whose TTVL 3-months post-transplant exceeded the currently proposed cutoff to predict infections within the first year post-transplant [6.2 log_10_] showed a trend towards a higher risk of being hospitalized with an infection in the following 9 months, albeit without being statistically significant (HR=1.642, *P*=0.07).

**Conclusions:**

Higher TTVL reflects the greater immunosuppressive burden in immunological high-risk patients receiving intensive immunosuppression. The choice of induction agent and intensified immunosuppressive maintenance therapy notably affects TTVL at 3 months post-transplant and beyond, necessitating careful consideration when interpreting and applying TTVL cutoffs to monitor immunocompetence post-transplant.

## 1 Introduction

Induction therapy—often involving a biologic agent like a lymphocyte-depleting agent or an interleukin-2 receptor antagonist (IL-2 RA)—is applied to modulate T-cell responses during antigen presentation at the time of transplantation, as oral maintenance immunosuppressants exhibit a delayed effect on immune cells (1). By reducing the rate of acute rejections and potentially lowering the need for other immunosuppressants such as calcineurin inhibitors or corticosteroids, induction therapy has been shown to augment the effectiveness of immunosuppressive therapy (2–4).

The choice of induction agent often mirrors the immunological risk pre-transplantation, with most centers favoring T-cell depleting therapy in medium to high immunological risk kidney transplant recipients (5). Annual data from the Organ Procurement and Transplantation Network (OPTN) and the Scientific Registry of Transplant Recipients (SRTR) report that induction immunosuppression was used in 92.1% of adult kidney transplants in 2022, with first-year post-transplant rejection rates at 7.0% for those receiving IL-2 RA, 6.0% for those given T-cell depleting agents, and 4.7% for those managed without induction therapy (6). In general, rates of clinical acute rejection in the first year post-transplant have been reported to range from 10 to 15%, while subclinical rejection occurs in about 5 to 15% of cases (7). Additionally, up to 40% of transplant recipients may experience subclinical inflammation – borderline changes – that are suspicious for T-cell mediated rejection (TCMR) but do not yet meet the BANFF criteria for rejection (7).

Although potent immunosuppression effectively lowers the risk of rejection, it also poses a considerable risk for infectious complications, which vary with the extent of immunosuppression and the different types of infectious exposures in the early perioperative and later post-transplant periods (8–11). While preventing rejections is crucial due to its significant contribution to death-censored graft failure (12), infectious complications rank as the second leading cause of death among kidney transplant recipients, following cardiovascular events (13, 14).

Therefore, determining the ideal net state of immunosuppression that balances the risks of rejection and infection is essential for improving post-transplant outcomes. Recently, torque teno virus load (TTVL) has been suggested as a biomarker to monitor immunocompetence following solid organ transplantation (15). Torque teno virus (TTV) is a non-pathogenic, highly prevalent virus that constitutes a significant proportion of the human blood virome (16). TTVL increases significantly with the initiation of immunosuppressive therapy but is largely unaffected by post-transplant antiviral prophylaxis and treatments, including CMV prophylaxis (16, 17). Previous studies have demonstrated a relationship between lower TTVL and an increased risk of rejection, as well as a correlation between higher TTVL and a greater risk of infections (18–22). Additionally, emerging evidence suggests that TTVL may be useful to track short-term changes in immunosuppressive therapy (23–26). Consequently, TTVL has been proposed as a potential tool for monitoring immunosuppressive therapy following transplantation and randomized controlled trials are currently underway to evaluate its utility in guiding immunosuppressive therapy post-transplantation (27). These trials include the TTV-guideIT trial, which focuses on managing immunosuppressive therapy in the first year post-transplant with first results expected in 2025 (28), as well as the TAOIST (TTV-guided mAnagement Of long-term ImmunosuppreSsion in kidney Transplantation) trial, which examines TTV-guided management of long-term immunosuppression following kidney transplantation. Although the evidence supporting TTVL as a tool to monitor immunocompetence expands (18–22), uncertainties remain regarding the impact of different induction agents on peak viral loads and the dynamics of viral loads in response to different immunosuppressive regimens after transplantation.

Yet, integrating monitoring TTVL into clinical routine requires a careful assessment of factors, such as induction therapy, that could influence peak viral load measurements to ensure the accurate interpretation of results. To address this issue, we conducted a retrospective analysis of TTVL in kidney transplant recipients who received different induction agents and immunosuppressive maintenance therapies, comparing outcomes and assessing infectious complications within this cohort.

## 2 Materials and methods

### 2.1 Study design

349 adult patients who underwent kidney transplantation at the Department of Nephrology, Heidelberg University Hospital between 2018 and 2021 were screened for this retrospective study. To evaluate the influence of induction therapy on TTVL, all 84 kidney transplant recipients who received anti-thymocyte globulin (ATG) induction therapy during that period were initially selected to be later matched to patients receiving an IL-2 RA as induction agent. Of these 84 patients receiving induction with ATG, 17 patients were excluded due to the following reasons: less than two blood samples during the observational period (N=13), simultaneous heart-kidney-transplantation (N=2), or no suitable match within the IL-2 RA group (N=2). To specifically characterize the effect of immunosuppressive therapy on TTVL, patients receiving induction therapy with ATG (N=67) were further subclassified as follows: those receiving rituximab for induction and higher tacrolimus target levels due to very high immunological risk (“ATG+Rituximab”; N=12), those with higher tacrolimus target levels (“ATG+high Tac”; N=18), those with standard tacrolimus target levels (“ATG+standard Tac”; N=21), and those receiving a simultaneous pancreas-kidney transplant (“ATG+SPK”; N=16). Importantly, ATG is typically administered at a low-dose of 1.5 mg/kg body weight before transplantation in Heidelberg, followed by additional doses based on the post-transplant lymphocyte count, usually not exceeding three post-operative administrations of ATG.

All of the 67 patients receiving ATG as induction therapy were matched with 67 patients receiving an IL-2 RA as induction agent in terms of age (± 5 years) and sex to address the known influence of age and sex on variations in TTVL (29, 30). Both groups were also matched based on donor modality to minimize any potential impact on graft function.

While the immunosuppressive regimen of the patients who received ATG always included tacrolimus, 29 (43%) of patients induced with IL-2 RA received cyclosporine A (CsA) instead and were analyzed separately. ABO incompatible living kidney transplants were excluded from the study due to the individuality in the choice of induction therapy.

**Figure 1:**
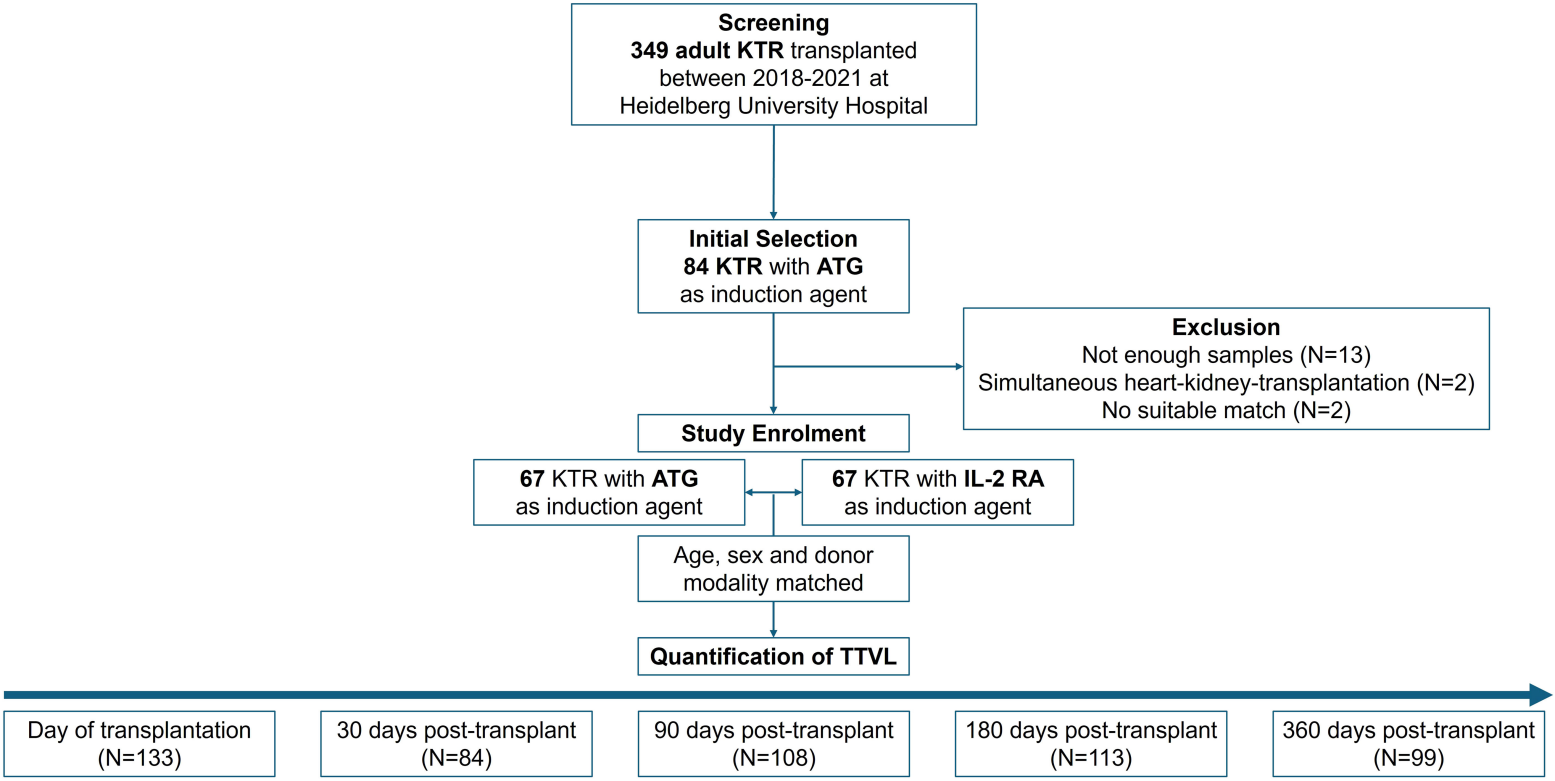
Study design and patient selection. Initially, 84 patients who received ATG were selected and later matched with patients receiving IL-2 RA induction therapy. Seventeen patients were excluded due to insufficient blood samples (N=13), simultaneous heart-kidney transplantation (N=2), or lack of suitable matches (N=2). The final cohort included 134 patients (67 in each group) matched for age (± 5 years), sex, and donor modality. TTVL was measured at transplantation and at 30-, 90-, 180-, and 360-days post-transplant. ATG, anti-thymocyte globulin; IL-2 RA, interleukin-2 receptor antagonist; KTR, kidney transplant recipients; N, Number; TTVL, torque teno virus load.

For all 134 kidney transplant recipients included into our study, TTVL was quantified at the time of transplantation and 30, 90, 180, and 360 days thereafter in a total of 537 samples. Study design is shown in **Figure 1**.

The study was approved by the University of Heidelberg ethics committee and conducted according to the Declaration of Helsinki. The clinical and research activities being reported are consistent with the Principles of the Declaration of Istanbul as outlined in the ‘Declaration of Istanbul on Organ Trafficking and Transplant Tourism’. The study is registered within the German Clinical Trials Register (DRKS00032849). The main goal of this analysis was to assess the impact of different induction agents and immunosuppressive maintenance therapies on (i) TTVL and (ii) complications in the first year post-transplant.

### 2.2 The Heidelberg algorithm for classifying immunological risk at transplantation

Immunosuppression is tailored based on the immunological risk at the time of transplantation (**Supplementary Table S1**). Patients classified as “low risk”—those without donor-specific antibodies (DSA) and with virtual panel reactive antibodies (vPRA) ≤ 30%—receive an interleukin-2 receptor antagonist (IL-2 RA; 20mg Basiliximab) as an induction agent, administered immediately before transplantation and on day 4 post-transplant. Additionally, these patients receive a triple immunosuppressive regimen consisting of a calcineurin inhibitor (CNI), mycophenolic acid (MPA), and corticosteroids (CS) with subsequent reduction. Tacrolimus is the preferred CNI since late 2018 at our center, with trough levels aiming at 8-10µg/ml (until week 6 post-transplant), 5-8µg/ml (until month 3), and 4-6µg/ml after the third month post-transplant.

Patients without DSAs but vPRA >30% are “intermediate low risk” and receive ATG as induction agent and the same triple immunosuppressive therapy afterwards. Patients with DSAs prior to transplantation (for retransplanted patients and/or patients with a positive Luminex antibody screen class I and II with an MFI ≥3000, or an MFI ≥ 5000 for all others) are defined as “intermediate high risk” and further receive plasmapheresis once pre-transplant and twice post-transplant.

Patients that are “high risk” are patients 1) with a calculated PRA >85%, 2) positive for anti-HLA class I and II (Luminex), 3) positive for anti-HLA class I (Luminex) and re-transplanted, and 4) positive for anti-HLA class II, retransplanted, and with a positive B-cell crossmatch. Those patients receive induction with ATG and rituximab in a dose of 1x375mg/m^2^. Additionally, plasmapheresis is performed once pre-transplant and at least six times post-transplantation until S-Creatinine levels are below 2mg/dl and DSAs are negative. The dosing for MPA (2x720mg mycophenolate sodium) and corticosteroids is the same for these patients, but tacrolimus trough levels aim higher with 11-15µg/ml (until day 14), 9-13µg/ml (until day 28), 7-11µg/ml (until month 6), 4-10µg/ml (until month 12), and 3-9µg/ml afterwards.

Patients with simultaneous pancreas kidney transplantation (SPK) always receive ATG as induction agent as per center standard. Tacrolimus trough levels are also maintained at higher doses, similar to those for patients at higher immunological risk.

### 2.3 Quantification of torque teno viral loads

TTVL was quantified using the Torque Teno Virus (TTV) R-Gene^®^ assay (BioMérieux, Marcy-l’Etoile, France), a real-time PCR assay that targets the TTV 5′ untranslated region (31). The dynamic range for quantification is from 250 to 10^9^ copies/mL. Each run includes a sensitivity, a negative, and a positive control and four quantification standards. The TTV R-Gene^®^ assay was developed and validated for quantifying TTVL in plasma and whole blood samples and is widely used for this purpose (32). We recently demonstrated that plasma and serum samples can be used interchangeably for TTVL quantification with the TTV R-Gene^®^ assay (24). Therefore, both serum and plasma samples were used in this study depending on availability. TTV DNA was extracted from samples using the QIAsymphony SP platform (QIAGEN, Venlo, Netherlands). PCR was then performed on a Light Cycler^®^ 480 Instrument II (Roche Diagnostics, Rotkreuz, Switzerland) following the manufacturer’s instructions. Viral load was measured using a standard curve, and samples with undetectable viral load were assigned a value of 0.01 copies/mL for analysis purposes, as previously done so by Fernández-Ruiz et al. (33). In total, 537 samples were analyzed, with a mean of 4 samples per patient. TTV was above the threshold of detection in 514 (96%) samples. Of the 23 samples where TTV was not detected, 19 (83%) were taken at the time of transplantation and thus before the start of immunosuppressive therapy.

### 2.4 Statistical analysis

Statistical analysis was performed using GraphPad Prism version 10.2.3 (GraphPad Software, San Diego, CA, United States) and statistical significance was assumed at a *P*-value <0.05. To compare TTVL between groups the Mann-Whitney *U* test was used. The unpaired t test was used for parametrical data. Comparisons among more than two different groups were made using the Kruskal–Wallis test followed by Dunn’s post-test. Spearman’s rho was calculated to correlate TTVL to cytomegalovirus (CMV) and BK virus (BKV) loads if CMV or BKV was detected by PCR at 30-, 90- 180-, or 360-days post-transplant when TTVL was quantified. Survival data is shown using Kaplan-Meier curves. Survival curves were compared using the Log-rank (Mantel-Cox) test and hazard ratios were calculated using the log-rank method. Quantitative data is presented as median with interquartile range (IQR) or as mean with standard deviation (SD).

## 3 Results

### 3.1 Study cohort

The clinical characteristics of the participants included in this study, analyzed overall and by type of induction therapy are shown in **Table 1**. The mean age (±SD) at transplantation was 46 (± 11) years, 48 (36%) of the patients were female. Evidently, among those receiving ATG as induction agent, re-transplantations were more frequent with 28 (42%) patients having undergone at least one prior transplantation, while only 3 (4%) of those receiving IL-2 RA induction had a prior transplantation. All patients received a triple immunosuppressive treatment with a calcineurin inhibitor, mycophenolic acid, and corticosteroids.

**Table 1:** Baseline characteristics of the study cohort.

Variable	All (N=134)	Induction with ATG (N=67)	Induction with IL-2 RA (N=67)
Number of samples, N	537	256	281
Female, N (%)	48 (36)	24 (36)	24 (36)
Age, mean ± SD	46 ± 11	46 ± 11	46 ± 11
Body mass index, mean ± SD	25.65 ± 4.86	25.94 ± 5.43	25.35 ± 4.22
Donor type, N (%) Deceased donor Living donor	122 (94) 8 (6)	63 (94) 4 (6)	63 (94) 4 (6)
Donor age, mean ± SD	52 ± 16	47 ± 17	57 ± 14
Prior transplantation, N(%)	31 (23)	28 (42)	3 (4)
Combined transplantation, N(%) Pancreas Liver	18 (13) 16 (12) 2 (1)	16 (24) 16 (24) -	2 (3) - 2 (3)
Baseline immunosuppressive regimen, N (%) Tacrolimus Cyclosporine A Mycophenolic acid Corticosteroids	105 (78) 29 (22) 134 (100) 134 (100)	67 (100) - 67 (100) 67 (100)	38 (57) 29 (43) 67 (100) 67 (100)
Renal pathology, N (%) Diabetes Vascular IgA nephropathy Other GN PKD Interstitial Others/Unknown	19 (14) 6 (4) 11 (8) 43 (32) 14 (10) 19 (14) 22 (16)	18 (27) 3 (4) 5 (7) 15 (22) 3 (4) 10 (15) 13 (19)	1 (1) 3 (4) 6 (9) 28 (42) 11 (16) 9 (13) 9 (13)
Complications, N (%) Rejection Infection BKVAN DCGF Death	26 (19) 91 (68) 10 (7) 2 (1) 3 (2)	14 (21) 45 (67) 5 (7) 1 (1) 3 (4)	12 (18) 46 (69) 5 (7) 1 (1) -
eGFR, mL/min, mean ± SD 90 days post Tx 180 days post Tx 360 days post Tx	52 ± 22 52 ± 23 53 ± 23	56 ± 25 55 ± 26 54 ± 26	48 ± 18 50 ± 19 51 ± 19
Lymphocytes, g/nL, mean ± SD 90 days post Tx 180 days post Tx 360 days post Tx	1.26 ± 1.18 1.47 ± 1.74 1.56 ± 1.00	1.00 ± 1.49 1.42 ± 2.46 1.44 ± 1.30	1.51 ± 0.69 1.51 ± 0.63 1.69 ± 0.50

ATG, anti-thymocyte globulin; BKVAN, BK virus-associated nephropathy; DCGF, death censured graft failure; eGFR, estimated glomerular filtration rate; GN, glomerulonephritis; Il-2 RA, interleukin-2 receptor antagonist; PKD, polycystic kidney disease; SD, standard deviation; Tx, transplantation.

### 3.2 Dynamics of torque teno virus load following transplantation

**Figure 2:**
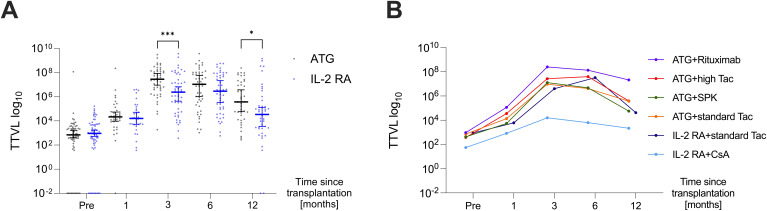
Dynamics of torque teno virus loads in the first-year post-transplant. **(A)** TTVL at various time points pre- and post-transplant (day 0, 30, 90, 180, and 360) in kidney transplant recipients receiving ATG (black) or IL-2 RA (blue) as induction therapy displayed as median ±95% confidence interval. **(B)** Median TTVL at various time points pre- and post-transplant (day 0, 30, 90, 180, 360) in patients receiving ATG induction therapy (i) with additional rituximab (“ATG+Rituximab”, N=12; purple curve), (ii) with higher tacrolimus target levels in high immunological risk settings (“ATG+high Tac”, N=18; red curve), (iii) for simultaneous pancreas-kidney transplantation (“ATG+SPK”, N=16; green curve), (iv) in intermediate immunological risk settings (“ATG+standard Tac”, N=21; orange curve). TTVL is also shown for patients receiving IL-2 RA induction with subsequent tacrolimus (“IL-2 RA+standard Tac”, N=38; navy blue curve) and IL-2 RA with subsequent cyclosporine A (“IL-2 RA+CsA”, N=29; light blue curve). For patients with standard tacrolimus (Tac), trough levels are aimed at 8-10 µg/ml until week 6 post-transplant, 5-8 µg/ml until month 3, and 4-6 µg/ml after the third month. In contrast, patients receiving high Tac have trough level targets of 11-15 µg/ml until day 14, 9-13 µg/ml until day 28, 7-11 µg/ml until month 6, 4-10 µg/ml until month 12, and 3-9 µg/ml thereafter. ATG, anti-thymocyte globulin; CsA, cyclosporine A; IL-2 RA, interleukin-2 receptor antagonist; SPK, simultaneous pancreas-kidney transplantation; Tac, tacrolimus; TTVL, torque teno virus load; *P < 0.05, ***P < 0.001.

TTVL was lowest at the time of transplantation with a median (IQR) of 6.95×10^2^ c/mL (2.48×10^2^–2.35×10^3^ c/mL) and 9.33×10^2^ c/mL (2.18×10^2^–4.97×10^3^ c/mL) in the ATG and IL-2 RA group, respectively, with no significant difference between both groups at baseline. Afterwards, TTVL steeply increased, with viral loads 30 days post-transplant at a median (IQR) of 2.15×10^4^ c/mL (7.06×10^3^–8.82×10^4^ c/mL) in the ATG group and 1.60×10^4^ c/mL (4.87×10^3^–1.10×10^5^ c/mL) in the IL-2 RA group (*P*=0.632). Ninety days after transplantation, viral loads reached their peak levels for all transplanted patients, with patients receiving ATG as induction agent having a significantly higher TTVL with a median (IQR) of 2.82×10^7^ c/mL (3.93×10^6^–1.30×10^8^ c/mL) compared to those receiving IL-2 RA as induction agent with a median (IQR) of 2.40×10^6^ c/mL (5.73×10^4^–2.60×10^7^ c/mL; *P*<0.001). TTVL remained elevated 180 days post-transplant with a median (IQR) of 1.09×10^7^ c/mL (3.24×10^5^–1.03×10^8^ c/mL) and 2.92×10^6^ c/mL (9.88×10^4^–5.89×10^7^ c/mL) in the ATG and IL-2 RA group, respectively (*P*=0.242). After 360 days, TTVL had decreased noticeably, but patients who received ATG as induction agent and were set to higher tacrolimus trough levels still exhibited higher viral loads than their IL-2 RA counterparts (median [IQR] 3.79×10^5^ c/mL [2.89×10^4^–1.60×10^7^ c/mL] vs. 3.42×10^4^ c/mL [2.39×10^3^–4.91×10^5^ c/mL], *P*=0.02; **Figure 2A**).

When analyzing the various immunosuppressive induction and maintenance therapies, significant differences in TTVL across the different groups were observed at three- and six-months post-transplant (*P*=0.003 and *P*=0.01, respectively, **Figure 2B**). Throughout all post-transplant time points, patients receiving additional rituximab for induction along with higher tacrolimus target levels (“ATG+Rituximab”; N=12) exhibited the highest TTVL (**Figure 2B**).

While all patients who received ATG as induction agent received tacrolimus as CNI, patients receiving IL-2 RA as induction agent received either tacrolimus (N=38) or cyclosporine A (N=29). These subgroups were compared individually to patients receiving ATG as induction agent, the results are shown in **Table 2**. Both subgroups within the IL-2 RA group, whether receiving tacrolimus or cyclosporine A, had significantly lower viral loads at 90 days post-transplant compared to patients who received ATG as induction agent. Patients induced with IL-2 RA whose CNI maintenance therapy was cyclosporine A also showed significantly lower TTV loads 180 and 360 days after transplantation compared to patients induced with ATG and receiving tacrolimus as immunosuppressive maintenance therapy (*P*=0.004 and *P*=0.04, respectively).

**Table 2:** Dynamics of torque teno virus loads among different immunosuppressive regimens.

Time point	ATG+Tac (N=67)	IL-2 RA+Tac (N=38)	*P* IL-2 RA+Tac *to* ATG+Tac	IL-2 RA+CsA (N=29)	*P* IL-2 RA+CsA *to* ATG+Tac	*P* IL-2 RA+CsA *to* IL-2 RA+Tac
	TTVL median (IQR)	TTVL median (IQR)		TTVL median (IQR)		
**at Tx**	6.95×10^2^ (2.47×10^2^–2.35×10^3^)	8.73×10^2^ (1.28×10^2^–2.67×10^3^)	0.96	1.46×10^3^ (3.96×10^2^–5.82×10^3^)	0.07	0.18
**30d**	2.47 ×10^4^ (8.05×10^3^–8.89×10^4^)	6.07×10^3^ (1.29×10^3^–8.41×10^4^)	0.16	2.17×10^4^ (6.92×10^3^–3.85×10^5^)	0.54	0.10
**90d**	3.17×10^7^ (4.14×10^6^–1.38×10^8^)	3.99×10^6^ (2.86×10^5^–8.49×10^7^)	0.02 (*)	4.10×10^5^ (1.89×10^4^–1.34×10^7^)	<0.001 (***)	0.12
**180d**	1.45×10^7^ (3.82×10^5^–1.05×10^8^)	3.25×10^7^ (1.19×10^6^–1.01×10^8^)	0.53	1.63×10^5^ (1.94×10^4^–3.57×10^6^)	0.004 (**)	<0.001 (***)
**360d**	3.49×10^5^ (2.89×10^4^–1.60×10^7^)	4.25×10^4^ (7.28×10^2^–1.33×10^6^)	0.09	5.72×10^4^ (4.17×10^3^–1.65×10^5^)	0.04 (*)	0.95

ATG, anti-thymocyte globulin; CsA, cyclosporin A; d, days post-transplant; Il-2 RA, interleukin-2 receptor antagonist; IQR, interquartile range; N, number; Tac, Tacrolimus; TTVL, torque teno virus load; TX, transplantation; *P < 0.05; **P < 0.01; ***P < 0.001.

Tacrolimus trough levels were generally lower in patients receiving IL-2 RA as induction agent compared to patients receiving ATG, albeit only being significantly different at 180 days post-transplant (10.8 ± 4.5µg/mL for patients in the ATG group and 7.5 ± 2.4µg/mL for patients in the IL-2 RA group, *P*=0.002; **Table 3**). We did not find any significant correlation between tacrolimus trough levels and TTVL (Spearman’s rho=0.04; *P*=0.54). As additional information, cyclosporine A trough levels are given in **Supplementary Table S2**.

**Table 3:** Tacrolimus trough levels for the study cohort.

Time point	ATG+Tac (N=67)	IL-2 RA+Tac (N=38)	*P* IL-2 RA+Tac *to* ATG+Tac
	Tac trough level [µg/mL] (mean ± SD)	Tac trough level [µg/mL] (mean ± SD)	
**30d**	9.5 (± 3.6)	9.2 (± 3.0)	0.62
**90d**	9.2 (± 3.0)	8.6 (± 2.1)	0.36
**180d**	10.8 (± 4.5)	7.5 (± 2.4)	0.002 (**)
**360d**	7.1 (± 2.7)	7.1 (± 2.4)	0.91

ATG, anti-thymocyte globulin; Il-2 RA, interleukin-2 receptor antagonist; N, number; SD, standard deviation; Tac, tacrolimus; **P < 0.01.

### 3.3 Rejections and infections within 1 year post-transplant

**Figure 3:**
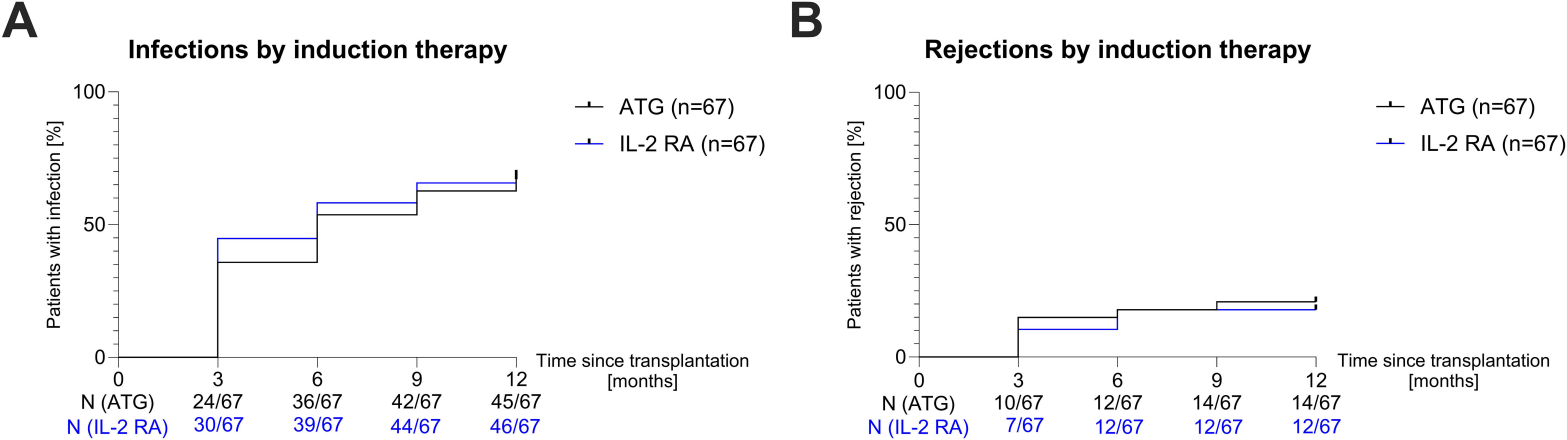
Hospitalization due to infection and rejection in year one post-transplant. **(A)** Survival curves showing the incidence of hospitalization due to infection in the first year post-transplant for patients receiving ATG (black curve) or IL-2 RA (blue curve) induction therapy in three-month intervals. **(B)** Survival curves showing the incidence of rejection events in the first year post-transplant for patients receiving ATG (black curve) or IL-2 RA (blue curve) induction therapy in three-month intervals. ATG, anti-thymocyte globulin; IL-2 RA, interleukin-2 receptor antagonist; N, number.

We explored whether the induction agent and the immunosuppressive maintenance regimen used influenced the incidence of complications, i.e. graft rejection or infection, in the first year post-transplant. Within the first year post-transplant, 45/67 (67%) patients who received induction with ATG were at least once hospitalized due to an infection, compared to 46/67 (69%) patients who received induction with IL-2 RA (Hazard Ratio [HR]=0.9292; *P*=0.6705; **Figure 3A**). There was also no significant difference in rejection events between the groups: 14/67 (21%) patients in the ATG group and 12/67 (18%) in the IL-2 RA group experienced at least one rejection in year one post-transplant (HR=1.183; *P*=0.6535; **Figure 3B**). Most of these rejections were classified as borderline changes suspicious for TCMR (23/26, 88%). Of the three patients with acute TCMR, two received induction therapy with IL-2 RA, while the other patient received induction with ATG.

**Figure 4:**
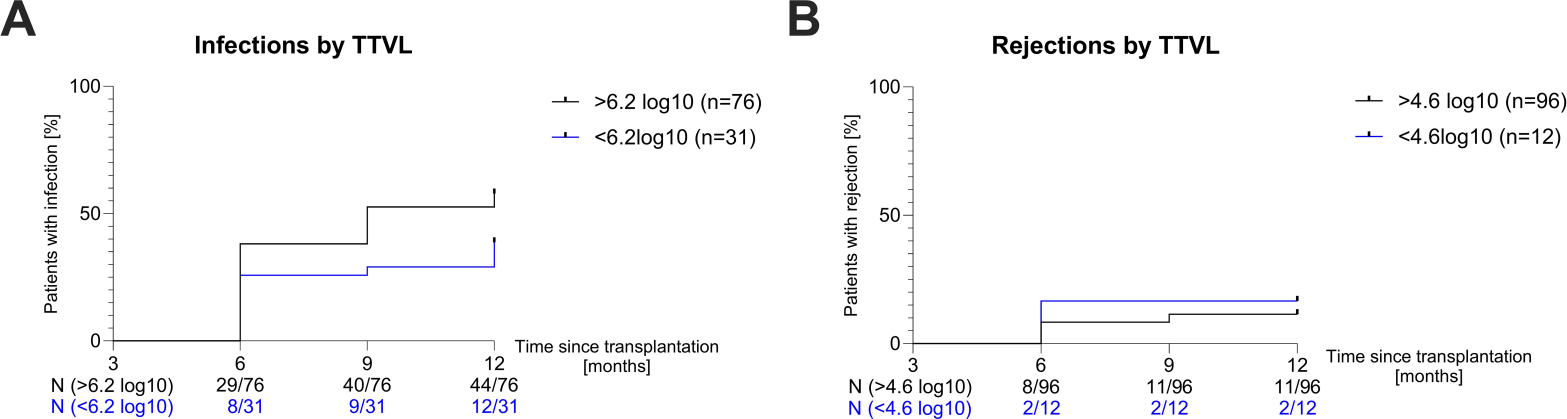
Risk of infection and rejection based on torque teno virus loads. **(A)** Survival curves showing the risk of hospitalization due to infection in the following nine months in patients with TTVL above (black curve) and below (blue curve) the proposed upper limit (6.2 log_10_) at 90 days post-transplant. **(B)** Survival curves showing the risk of rejection in the following nine months in patients with TTVL above (black curve) and below (blue curve) the proposed lower limit (4.6 log_10_) at 90 days post-transplant. N, number; TTVL, torque teno virus load.

Afterwards, we investigated whether patients whose TTVL 3 months post-transplant was outside the currently proposed range of 4.6–6.2 log_10_ c/mL to guide immunosuppressive therapy (28) were at higher risk of rejection or infection in the following 9 months. Patients whose TTVL 3 months post-transplant exceeded the proposed cutoff of 6.2 log_10_ c/mL (N=76) had a higher risk of being hospitalized with an infection in the following 9 months, albeit without being statistically significant (HR=1.642, *P*=0.07; **Figure 4A**). Similarly, patients below the lower cutoff of 4.6 log_10_ c/mL (N=12) showed a higher risk of rejection (HR=1.486) but this again failed to reach statistical significance (*P*=0.59; **Figure 4B**).

Neither risks for infections nor rejections were significantly increased when applying the cutoffs separately in the ATG and IL-2 RA group (**Supplementary Figure S1**). There were also no significant differences in the risks of either infection or rejection between 6 to 12 months after transplantation when the cutoffs were applied to TTVL at 6 months post-transplant (**Supplementary Figure S2**).

### 3.4 Infection sites and pathogens

**Figure 5:**
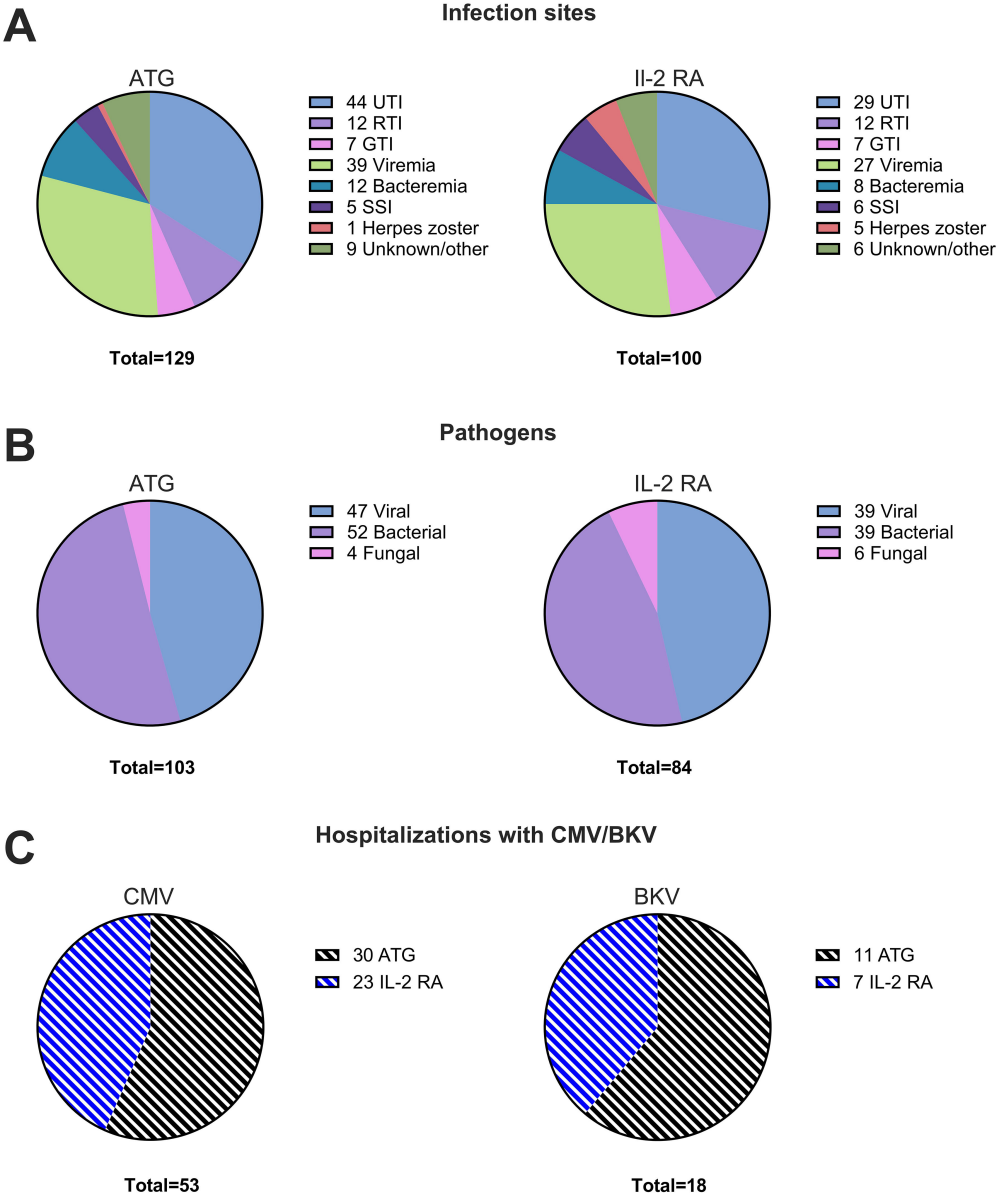
Infection sites and pathogens in year one post-transplant. **(A)** Distribution of infection sites in kidney transplant recipients within the first year post-transplant stratified according to induction therapy for patients receiving ATG (left panel) or IL-2 RA (right panel). **(B)** Types of pathogens identified in infections within the first year post-transplant for patients receiving induction therapy with ATG (left panel) or IL-2 RA (right panel). **(C)** Hospitalizations with CMV (left panel) and BKV (right panel) in patients receiving induction with ATG (dashed black) or IL-2 RA (dashed blue). ATG, anti-thymocyte globulin; BKV; BK virus; CMV, cytomegalovirus; GTI, gastrointestinal tract infection; IL-2 RA, interleukin-2 receptor antagonist; RTI, respiratory tract infection; SSI, surgical site infection; UTI, urinary tract infection.

With 91/134 (68%) of all kidney transplant recipients in our study contracting at least one infection that required hospitalization in the first year post-transplant, we analyzed which infection sites were most common, identified the responsible pathogen, and stratified according to induction therapy. Specifically, there were 129 hospitalizations due to infections in patients who received induction therapy with ATG, compared to 100 cases in patients who received IL-2 RA as induction therapy. Notably, the numbers include recurrent infections. The most common infection site for bacterial infections was the urinary tract (N=73 in total; 44 in the ATG and 29 in the IL-2 RA group). Viremia was also registered frequently (N=66 in total; 39 in the ATG and 27 in the IL-2 RA group). Furthermore, we registered 24 respiratory tract infections, 20 cases of bacteremia, 14 gastrointestinal tract infections, 11 surgical site infections, 6 cases of herpes zoster, and 15 other infections, most with unknown infection focus (**Figure 5A**).

When a pathogen was detected, it was more commonly viral (N=86 in total; 47 in the ATG and 39 in the IL-2 RA group) or bacterial (N=91 in total; 52 in the ATG and 39 in the IL-2 RA group), with some instances of fungal infections (N=10 in total; 4 in the ATG and 6 in the IL-2 RA group; **Figure 5B**).

Viral infections necessitating hospitalization comprised viremia with CMV (N=53 in total; 30 in the ATG and 23 in the IL-2 RA group) and BKV (N=18 in total; 11 in the ATG and 7 in the IL-2 RA group; **Figure 5C**). Viremia was defined as CMV and BKV loads over 400 copies/mL. Notably, BKV cases were more frequent (31 patients in total) but required hospitalization only in instances of unresolved BK viremia and suspected BK nephropathy, which needed verification by graft biopsy.

The TTVL of patients with BK viremia correlated significantly with BKV loads (Spearman’s rho= 0.38; *P*=0.004). However, there was no significant correlation between CMV loads and TTVL in patients with CMV viremia (**Supplementary Table S3**). Correlations were performed only for samples with available BKV or CMV results. This analysis included BKV and CMV loads of patients hospitalized with BKV or CMV and those of patients who exhibited BKV or CMV viremia at routine follow-up.

Infection sites and pathogens in all transplant recipients over time are displayed in **Supplementary Figure S3** while **Supplementary Figure S4** shows the specific pathogens detected.

## 4 Discussion

Our study provides novel insights into the differential impact of induction therapies and differences in immunosuppressive maintenance therapies on TTVL in kidney transplant recipients.

The first finding is that patients receiving ATG as induction agent exhibit significantly higher TTVL at peak viral loads three months post-transplant compared to those receiving IL-2 RA. These results suggest that ATG induction exerts a more profound and prolonged influence on TTV replication, likely due to its potent immunosuppressive effects (34, 35), which may sustain higher viral replication. Interestingly, Focosi et al. found that within 7-15 days post-transplant, patients receiving ATG induction had lower TTVL than those receiving IL-2 RA, likely due to the immediate depletion of TTV replication-competent peripheral mononuclear cells (36). However, one month post-transplant, there were no significant differences in TTVL between the two groups, aligning with our findings and other studies (33, 37). As lymphocyte counts were not significantly different between patients receiving ATG and those receiving IL-2 RA at three months post-transplant within our study, the higher peak viral loads three months post-transplant likely reflect the strong degree of immunosuppression exerted by ATG on the immune system, not necessarily the count of replication-competent cells. Notably, patients receiving rituximab additionally as induction agent displayed even higher viral loads 3 months post-transplant, with loads significantly elevated compared to those of patients receiving ATG only, implying a cumulative effect of immunosuppression on TTVL. A strong effect on TTVL by rituximab was previously shown in patients with rheumatoid arthritis (38) and in kidney transplant recipients treated with rituximab (39), with the latter study even suggesting that rituximab has a stronger impact on TTVL than ATG.

Secondly, our results show that patients who received ATG as an induction agent maintain higher viral loads subsequently, likely reflecting higher cumulative immunosuppression to minimize the risk for rejection in these pre-sensitized patients. Admittedly, our data cannot specify to what extent rituximab, ATG or higher tacrolimus target levels each contribute to the higher TTVL, yet they do suggest a cumulative effect of these different immunosuppressants. Additionally, treatment for rejection also influences TTVL, potentially explaining the unexpected further increase in TTVL observed in the “IL-2 RA+standard Tac” group 6 months post-transplant (**Figure 2B**). While our study, consistent with others, did not find a correlation between TTVL and mean tacrolimus blood levels (22, 40), evidence is strong that TTVL reflects the overall net state of immunosuppression in transplanted patients (27, 41). Interestingly, our results show higher TTVL in patients on tacrolimus compared to those on cyclosporine A from 3 months post-transplant onward, most likely reflecting the stronger immunosuppressive effect of tacrolimus, making it the preferred CNI post-transplant. This aligns with findings by Görzer et al., who demonstrated significantly lower TTVL in lung transplant recipients treated with cyclosporine A compared to those receiving tacrolimus (42).

Third, when applying the suggested TTVL range of 4.6–6.2 log_10_ to minimize rejections and infections in kidney transplant recipients (28), our findings indicate that patients with a TTVL above 6.2 log_10_ had a higher, though not significant, risk of infection-related hospitalization. Similarly, patients with a TTVL below 4.6 log_10_ were more prone to experience rejection, albeit not reaching statistical significance. Three months post-transplant, only 6 patients who received ATG induction were below the proposed upper limit of 6.2log_10_, compared to 25 patients in the IL-2 RA group. Despite higher TTVL, ATG recipients did not show an increased risk of hospitalization for infections compared to IL-2 RA recipients, suggesting that a higher TTVL in ATG recipients does not necessarily translate into clinical complications. Additionally, 11 patients experienced rejections despite being above the proposed 4.6 log_10_ threshold to suspect rejection (**Supplementary Figure S1**). While evidence supporting TTVL to predict rejections is strong (15), our data emphasizes that current cutoffs evidently need adjustments for predicting rejections in pre-sensitized patients receiving T-cell depleting therapy and potentially higher doses of immunosuppressive maintenance therapy. Of note, the current cutoffs to guide immunosuppressive therapy were predominantly established in low-risk transplant recipients, and ongoing trials using TTVL to dose immunosuppression exclude those on T-cell depleting therapy (28). Our data supports the necessity for further research on TTVL, particularly involving patients receiving more intense immunosuppression.

Our previous research indicated that patients diagnosed with BK virus nephropathy (BKVAN) exhibit significantly elevated TTVL compared to those with other histopathological diagnoses, and that BK virus loads correlate positively with TTVL (23). This study confirms these findings, consistent with other investigations (20, 43, 44), highlighting that higher TTVL reflects increased immunosuppressive burden, potentially promoting BK virus replication and increasing the risk of BKVAN development. Similarly, Eder et al. only recently demonstrated that ABO incompatible transplant recipients had significantly higher TTVL and a higher incidence of biopsy-proven BKVAN compared to recipients of HLA incompatible or immunological low-risk transplants (39).

Lastly, the observed dynamics in TTVL, with a steep increase post-transplant, peaking at 90 days, and subsequent decline, are consistent with previous studies (33, 43, 45), underscoring the robustness of TTVL as a biomarker for monitoring immunocompetence in kidney transplant recipients. Previously, we already demonstrated, that TTVL reflects short-term changes in immunosuppressive medication such as pausing mycophenolate (24), but that adjustments in immunosuppression, such as corticosteroid pulse therapy in response to rejection or switching to an mTOR inhibitor in patients with BKVAN, only affect TTVL about 30 days afterwards (23). Similarly, Regele et al. only recently demonstrated that changes in TTVL become noticeable only 2 months after tacrolimus dose adaptation (25). These findings raise doubts about using TTVL as a tool to timely dose immunosuppression. Current evidence, including our findings, suggests that TTVL may be better suited for assessing immunocompetence rather than guiding dosage of immunosuppressants, given the delayed impact of dose adjustments on TTVL. Additionally, the variability across centers in patient characteristics, immunosuppressive protocols, and anti-infective strategies (46), along with confounding factors affecting TTVL (29, 47, 48), may complicate establishing universal TTVL cutoffs.

Our study has some limitations: the retrospective design inherently limits establishing definitive causal relationships between induction therapy, immunosuppressive maintenance therapy, TTVL, and clinical outcomes. Therefore, we are unable to conclusively determine to which extent each variable contributed to the variations in TTVL. Notably, the higher TTVL observed in patients receiving induction therapy with ATG may also partly be explained by the fact that 43% of those who received IL-2 RA as induction therapy were maintained on cyclosporine A, while all patients with ATG induction were maintained on tacrolimus, a more potent immunosuppressant than cyclosporine A. Another limitation to our study is the single-center design that restricts the generalizability of the findings, as practices and patient populations vary across different institutions. Although the Heidelberg algorithm for immunosuppressive therapy post-transplantation is based on evidence mostly derived from findings of the Collaborative Transplant Study (www.ctstransplant.org) (49–52), incorporating the Predicted Indirectly ReCognizable HLA Epitopes algorithm (PIRCHE-II) and knowledge on donor epitope-specific HLA-antibodies could further refine immunological risk stratification before transplantation (53, 54). However, since these assessments are not yet integrated into our clinical practice, they were not included in this analysis. Additionally, the relatively low number of more severe rejection events, with 23/26 (89%) rejections being categorized as Borderline lesions, limits the statistical power to detect significant differences in between groups regarding rejection rates and complicates the interpretation of TTVL within this context. Finally, while efforts were made to match patients in terms of age and sex, other confounding factors on TTVL, such as CMV serostatus, and also potentially unknown factors were possibly not controlled entirely, which might influence the interpretation of results.

In conclusion, our study confirms that TTVL effectively mirrors the net state of immunosuppression in kidney transplant recipients across various induction and immunosuppressive maintenance therapies. Our findings underscore the necessity to individualize TTVL monitoring, considering the specific induction and immunosuppressive maintenance therapies. Future multicenter studies are essential to validate these observations and address the complexities involved in managing immunosuppression post-transplant.

## Data Availability

The raw data supporting the conclusions of this article will be made available by the authors, without undue reservation.

## Supplementary material

Supplementary Table 1 Immunological risk-class and corresponding immunosuppressive therapy according to the Department of Nephrology, Heidelberg University Hospital.

Supplementary Table 2 Cyclosporine A trough levels for the study cohort.

Supplementary Table 3 Correlation of torque teno virus load to cytomegalovirus and BK virus loads.

Supplementary Figure 1 Infection and rejection risk stratified by induction therapy and torque teno virus load.

Supplementary Figure 2 Infection and rejection risk according to 6-month torque teno virus load.

Supplementary Figure 3 Infection sites and pathogens in the first year post-transplant.

Supplementary Figure 4 Specific pathogens detected in the first year post-transplant.

## References

[1] Eckardt K-U Kasiske BL Zeier MG. Special issue: KDIGO clinical practice guideline for the care of kidney transplant recipients. Am J Transplant. (2009) 9:S1–S155. doi: 10.1111/j.1600-6143.2009.02834.x 19845597

[2] Webster AC Playford EG Higgins G Chapman JR Craig JC. Interleukin 2 receptor antagonists for renal transplant recipients&colon; a meta-analysis of randomized trials1. Transplantation. (2004) 77:166–76. doi: 10.1097/01.tp.0000109643.32659.c4 14742976

[3] Hill P Cross NB Barnett ANR Palmer SC Webster AC. Polyclonal and monoclonal antibodies for induction therapy in kidney transplant recipients. Cochrane Database Syst Rev. (2017) 2017:CD004759. doi: 10.1002/14651858.cd004759.pub2 PMC6464766 28073178

[4] Malvezzi P Jouve T Rostaing L. Induction by anti-thymocyte globulins in kidney transplantation: a review of the literature and current usage. J Nephropathol. (2015) 4:110–5. doi: 10.12860/jnp.2015.21 PMC4596294 26457257

[5] Furian L Bestard O Budde K Cozzi E Diekmann F Mamode N. European consensus on the management of sensitized kidney transplant recipients: A delphi study. Transpl Int. (2024) 37:12475. doi: 10.3389/ti.2024.12475 38665475 PMC11043529

[6] Lentine KL Smith JM Lyden GR Miller JM Dolan TG Bradbrook K. OPTN/SRTR 2022 annual data report: kidney. Am J Transplant. (2024) 24:S19–S118. doi: 10.1016/j.ajt.2024.01.012 38431360

[7] Hariharan S Israni AK Danovitch G. Long-term survival after kidney transplantation. N Engl J Med. (2021) 385:729–43. doi: 10.1056/nejmra2014530 34407344

[8] Fishman JA. Infection in organ transplantation. Am J Transplant. (2017) 17:856–79. doi: 10.1111/ajt.14208 28117944

[9] Agrawal A Ison MG Danziger-Isakov L. Long-term infectious complications of kidney transplantation. Clin J Am Soc Nephrol. (2021) 17:CJN.15971020. doi: 10.2215/cjn.15971020 PMC8823942 33879502

[10] Sommerer C Schröter I Gruneberg K Schindler D Behnisch R Morath C. Incidences of infectious events in a renal transplant cohort of the german center of infectious diseases (DZIF). Open Forum Infect Dis. (2022) 9:ofac243. doi: 10.1093/ofid/ofac243 35855001 PMC9280327

[11] van Delden C Stampf S Hirsch HH Manuel O Meylan P Cusini A. Burden and timeline of infectious diseases in the first year after solid organ transplantation in the swiss transplant cohort study. Clin Infect Dis. (2020) 71:e159–69. doi: 10.1093/cid/ciz1113 PMC7583409 31915816

[12] Gaston RS Fieberg A Helgeson ES Eversull J Hunsicker L Kasiske BL. Late graft loss after kidney transplantation: is “Death with function” Really death with a functioning allograft? Transplantation. (2019) 104:1483–90. doi: 10.1097/tp.0000000000002961 31568212

[13] Kinnunen S Karhapää P Juutilainen A Finne P Helanterä I. Secular trends in infection-related mortality after kidney transplantation. Clin J Am Soc Nephrol. (2018) 13:755–62. doi: 10.2215/cjn.11511017 PMC5969482 29622669

[14] Ying T Shi B Kelly PJ Pilmore H Clayton PA Chadban SJ. Death after kidney transplantation: an analysis by era and time post-transplant. J Am Soc Nephrol. (2020) 31:2887–99. doi: 10.1681/asn.2020050566 PMC7790214 32908001

[15] Jaksch P Görzer I Puchhammer-Stöckl E Bond G. Integrated immunologic monitoring in solid organ transplantation: the road toward torque teno virus-guided immunosuppression. Transplantation. (2022) 106:1940–51. doi: 10.1097/tp.0000000000004153 PMC9521587 35509090

[16] De Vlaminck I Khush KK Strehl C Kohli B Luikart H Neff NF. Temporal response of the human virome to immunosuppression and antiviral therapy. Cell. (2013) 155:1178–87. doi: 10.1016/j.cell.2013.10.034 PMC4098717 24267896

[17] Moen EM Sagedal S Bjøro K Degré M Opstad PK Grinde B. Effect of immune modulation on TT virus (TTV) and TTV-like-mini-virus (TLMV) viremia. J Méd Virol. (2003) 70:177–82. doi: 10.1002/jmv.10356 12629661

[18] Strassl R Schiemann M Doberer K Görzer I Puchhammer-Stöckl E Eskandary F. Quantification of torque teno virus viremia as a prospective biomarker for infectious disease in kidney allograft recipients. J Infect Dis. (2018) 218:1191–9. doi: 10.1093/infdis/jiy306 PMC6490304 30007341

[19] Strassl R Doberer K Rasoul-Rockenschaub S Herkner H Görzer I Kläger JP. Torque teno virus for risk stratification of acute biopsy-proven alloreactivity in kidney transplant recipients. J Infect Dis. (2019) 219:1934–9. doi: 10.1093/infdis/jiz039 PMC6534191 30668796

[20] Doberer K Schiemann M Strassl R Haupenthal F Dermuth F Görzer I. Torque teno virus for risk stratification of graft rejection and infection in kidney transplant recipients—A prospective observational trial. Am J Transplant. (2020) 20:2081–90. doi: 10.1111/ajt.15810 PMC7496119 32034850

[21] Doberer K Haupenthal F Nackenhorst M Bauernfeind F Dermuth F Eigenschink M. Torque teno virus load is associated with subclinical alloreactivity in kidney transplant recipients: A prospective observational trial. Transplantation. (2021) 105:2112–8. doi: 10.1097/tp.0000000000003619 PMC8376270 33587432

[22] Schiemann M Puchhammer-Stöckl E Eskandary F Kohlbeck P Rasoul-Rockenschaub S Heilos A. Torque teno virus load—Inverse association with antibody-mediated rejection after kidney transplantation. Transplantation. (2017) 101:360–7. doi: 10.1097/tp.0000000000001455 PMC5268087 27525643

[23] Reineke M Morath C Speer C Rudek M Bundschuh C Klein JAF. Dynamics of torque teno virus load in kidney transplant recipients with indication biopsy and therapeutic modifications of immunosuppression. Front Med. (2024) 11:1337367. doi: 10.3389/fmed.2024.1337367 PMC10847215 38327708

[24] Benning L Reineke M Bundschuh C Klein JAF Kühn T Zeier M. Quantification of torque teno virus load to monitor short-term changes in immunosuppressive therapy in kidney transplant recipients. Transplantation. (2023) 107(12):e363-9. doi: 10.1097/tp.0000000000004816 37798825

[25] Regele F Haupenthal F Doberer K Görzer I Kapps S Strassl R. The kinetics of Torque Teno virus plasma load following calcineurin inhibitor dose change in kidney transplant recipients. J Méd Virol. (2024) 96:e29554. doi: 10.1002/jmv.29554 38511586

[26] Regele F Heinzel A Hu K Raab L Eskandary F Faé I. Stopping of mycophenolic acid in kidney transplant recipients for 2 weeks peri-vaccination does not increase response to SARS-coV-2 vaccination—A non-randomized, controlled pilot study. Front Med. (2022) 9:914424. doi: 10.3389/fmed.2022.914424 PMC9226446 35755078

[27] Kapps S Haupenthal F Bond G. Torque Teno virus-guided monitoring of immunosuppressive therapy. Nephrol Dial Transplant. (2024) gfae149. doi: 10.1093/ndt/gfae149 PMC11596088 38925652

[28] Haupenthal F Rahn J Maggi F Gelas F Bourgeois P Hugo C. A multicenter, patient- and assessor-blinded, non-inferiority, randomized and controlled phase II trial to compare standard and torque teno virus-guided immunosuppression in kidney transplant recipients in the first year after transplantation: TTVguideIT. Trials. (2023) 24:213. doi: 10.1186/s13063-023-07216-0 36949445 PMC10032258

[29] Brassard J Gagné M-J Leblanc D Poitras É Houde A Boras VF. Association of age and gender with Torque teno virus detection in stools from diarrheic and non-diarrheic people. J Clin Virol. (2015) 72:55–9. doi: 10.1016/j.jcv.2015.08.020 26401905

[30] Haloschan M Bettesch R Görzer I Weseslindtner L Kundi M Puchhammer-Stöckl E. TTV DNA plasma load and its association with age, gender, and HCMV IgG serostatus in healthy adults. AGE. (2014) 36:9716. doi: 10.1007/s11357-014-9716-2 25284090 PMC4185385

[31] Focosi D Antonelli G Pistello M Maggi F. Torquetenovirus: the human virome from bench to bedside. Clin Microbiol Infect. (2016) 22:589–93. doi: 10.1016/j.cmi.2016.04.007 27093875

[32] Kulifaj D Durgueil-Lariviere B Meynier F Munteanu E Pichon N Dubé M. Development of a standardized real time PCR for Torque teno viruses (TTV) viral load detection and quantification: A new tool for immune monitoring. J Clin Virol. (2018) 105:118–27. doi: 10.1016/j.jcv.2018.06.010 29957546

[33] Fernández-Ruiz M Albert E Giménez E Ruiz-Merlo T Parra P López-Medrano F. Monitoring of alphatorquevirus DNA levels for the prediction of immunosuppression-related complications after kidney transplantation. Am J Transplant. (2019) 19:1139–49. doi: 10.1111/ajt.15145 30346659

[34] Halloran PF. Immunosuppressive drugs for kidney transplantation. N Engl J Med. (2004) 351:2715–29. doi: 10.1056/nejmra033540 15616206

[35] Bamoulid J Staeck O Crépin T Halleck F Saas P Brakemeier S. Anti-thymocyte globulins in kidney transplantation: focus on current indications and long-term immunological side effects. Nephrol Dial Transplant. (2017) 32:1601–8. doi: 10.1093/ndt/gfw368 27798202

[36] Focosi D Macera L Boggi U Nelli LC Maggi F. Short-term kinetics of torque teno virus viraemia after induction immunosuppression confirm T lymphocytes as the main replication-competent cells. J Gen Virol. (2015) 96:115–7. doi: 10.1099/vir.0.070094-0 25304651

[37] Handala L Descamps V Morel V Castelain S François C Duverlie G. No correlation between Torque Teno virus viral load and BK virus replication after kidney transplantation. J Clin Virol. (2019) 116:4–6. doi: 10.1016/j.jcv.2019.03.018 30986626

[38] Studenic P Bond G Kerschbaumer A Bécède M Pavelka K Karateev D. Torque Teno Virus quantification for monitoring of immunomodulation with biologic compounds in the treatment of rheumatoid arthritis. Rheumatology. (2021) 61:2815–25. doi: 10.1093/rheumatology/keab839 34792562

[39] Eder M Schrag TA Havel EF Kainz A Omic H Doberer K. Polyomavirus nephropathy in ABO blood group-incompatible kidney transplantation: torque teno virus and immunosuppressive burden as an approximation to the problem. Kidney Int Rep. (2024) 9:1730–41. doi: 10.1016/j.ekir.2024.04.003 PMC11184242 38899213

[40] Cañamero L Benito-Hernández A González E Escagedo C Rodríguez-Vidriales M García-Saiz M del M. Torque Teno Virus Load Predicts Opportunistic Infections after Kidney Transplantation but Is Not Associated with Maintenance Immunosuppression Exposure. Biomedicines. (2023) 11:1410. doi: 10.3390/biomedicines11051410 37239081 PMC10216334

[41] Eskandary F Bond G Mohan K. Torque teno virus-guided immunosuppression in kidney transplantation: expanding the application. Kidney Int Rep. (2024) 9:1568–70. doi: 10.1016/j.ekir.2024.04.057 PMC11184399 38899192

[42] Görzer I Haloschan M Jaksch P Klepetko W Puchhammer-Stöckl E. Plasma DNA levels of Torque teno virus and immunosuppression after lung transplantation. J Hear Lung Transplant. (2014) 33:320–3. doi: 10.1016/j.healun.2013.12.007 24559947

[43] Solis M Velay A Gantner P Bausson J Filipputtu A Freitag R. Torquetenovirus viremia for early prediction of graft rejection after kidney transplantation. J Infect. (2019) 79:56–60. doi: 10.1016/j.jinf.2019.05.010 31100359

[44] Fernández-Ruiz M Albert E Giménez E Rodríguez-Goncer I Andrés A Navarro D. Early kinetics of Torque Teno virus DNA load and BK polyomavirus viremia after kidney transplantation. Transpl Infect Dis. (2020) 22:e13240. doi: 10.1111/tid.13240 31883425

[45] Querido S Martins C Gomes P Pessanha MA Arroz MJ Adragão T. Kinetics of torque teno virus viral load is associated with infection and *de novo* donor specific antibodies in the first year after kidney transplantation: A prospective cohort study. Viruses. (2023) 15:1464. doi: 10.3390/v15071464 37515152 PMC10384556

[46] Redondo N Navarro D Aguado JM Fernández-Ruiz M. Viruses, friends, and foes: The case of Torque Teno Virus and the net state of immunosuppression. Transpl Infect Dis. (2022) 24:e13778. doi: 10.1111/tid.13778 34933413

[47] Cebriá-Mendoza M Beamud B Andreu-Moreno I Arbona C Larrea L Díaz W. Human anelloviruses: influence of demographic factors, recombination, and worldwide diversity. Microbiol Spectr. (2023) 11:e04928–22. doi: 10.1128/spectrum.04928-22 PMC10269794 37199659

[48] Herz CT Kulterer OC Kulifaj D Gelas F Franzke B Haupenthal F. Obesity is associated with a higher Torque Teno viral load compared to leanness. Front Endocrinol. (2022) 13:962090. doi: 10.3389/fendo.2022.962090 PMC9554490 36246898

[49] Morath C Beimler J Opelz G Ovens J Scherer S Schmidt J. An integrative approach for the transplantation of high-risk sensitized patients. Transplantation. (2010) 90:645–53. doi: 10.1097/tp.0b013e3181ea3985 20671598

[50] Süsal C Seidl C Schönemann C Heinemann FM Kauke T Gombos P. Determination of unacceptable HLA antigen mismatches in kidney transplant recipients: recommendations of the German Society for Immunogenetics. Tissue Antigens. (2015) 86:317–23. doi: 10.1111/tan.12682 26467895

[51] Süsal C Döhler B Opelz G. Presensitized kidney graft recipients with HLA class I and II antibodies are at increased risk for graft failure: A Collaborative Transplant Study report. Hum Immunol. (2009) 70:569–73. doi: 10.1016/j.humimm.2009.04.013 19375472

[52] Süsal C Opelz G. Kidney graft failure and presensitization against HLA Class I and Class II antigens1. Transplantation. (2002) 73:1269–73. doi: 10.1097/00007890-200204270-00014 11981420

[53] Unterrainer C Döhler B Niemann M Lachmann N Süsal C. Can PIRCHE-II matching outmatch traditional HLA matching? Front Immunol. (2021) 12:631246. doi: 10.3389/fimmu.2021.631246 33717167 PMC7952296

[54] Kardol-Hoefnagel T Senejohnny DM Kamburova EG Wisse BW Reteig L Gruijters ML. Determination of the clinical relevance of donor epitope-specific HLA-antibodies in kidney transplantation. HLA. (2024) 103:e15346. doi: 10.1111/tan.15346 38239046

