# Supplementary material for: Impact of induction agents and maintenance immunosuppression on torque teno virus loads and year-one complications after kidney transplantation
Source: Front Immunol. 2024 Nov 13;15:1492611. doi: 10.3389/fimmu.2024.1492611 (PMC11599233; doi:10.3389/fimmu.2024.1492611)
Supplement: Supplementary Table 1 — Immunological risk-class and corresponding immunosuppressive therapy according to the Department of Nephrology, Heidelberg University Hospital. [file DataSheet1.pdf]

Supplementary Material

Table of Contents

Supplementary Table S1 Immunological risk-class and corresponding immunosuppressive therapy according to the Department of Nephrology, Heidelberg University Hospital

Supplementary Table S2 Cyclosporine A trough levels for the study cohort

Supplementary Table S3 Correlation of torque teno virus load to cytomegalovirus and BK virus loads

Supplementary Figure S1 Infection and rejection risk stratified by induction therapy and torque teno virus load

Supplementary Figure S2 Infection and rejection risk according to 6-month torque teno virus load

Supplementary Figure S3 Infection sites and pathogens in the first year post-transplant

Supplementary Figure S4 Specific pathogens detected in the first year post-transplant

Supplementary Table S1 Immunological risk-class and corresponding immunosuppressive therapy according to the Department of Nephrology, Heidelberg University Hospital

Classification of Immunological Risk Categories

Criteria	cPRA>85%	Luminex AB Screen Class I and II positive	Luminex AB Screen Class I positive and re-transplanted	Luminex AB Screen Class II positive and retransplanted and positive B-Cell-XM
Positive for **one** of the above criteria	**YES** → **High Risk**			
	**NO**			

None of the above criteria:
Differentiation between the following constellations:

DSA positive (but no 'unacceptables'*)	No DSA but vPRA >30%	No DSA and vPRA ≤ 30%
**intermediate high risk**	**intermediate low risk**	**low risk**

* unaccabtables cut-off MFI :

- For re-transplanted patients and/or Luminex Ab Screen Class I and II positive ≥ 3000
- For all others ≥ 5000

AB antibody; cPRA, calculated panel reactive antibodies; DSA, donor-specific antibodies; MFI, mean fluorescence intensity; vPRA, virtual panel reactive antibodies; XM, cross-match

Immunosuppressive Maintenance Therapy

high risk	intermediate high risk	intermediate low risk	low risk
ATG (+Rituximab)<br>Tacrolimus, EC-MPS, Steroids<br>PPH 1x pre-transplant and ≥6x post-transplant (until S-Creatinine <2 mg/dL and DSA negative)	ATG<br>Tacrolimus, EC-MPS, Steroids<br>PPH 1x pre-transplant and 2x post-transplant	ATG<br>Tacrolimus, EC-MPS, Steroids	IL-2 RA<br>Tacrolimus**, EC-MPS, Steroids<br><br>***For immunological low risk patients, Tacrolimus is the preferred calcineurin inhibitor since 2018 at our center.*

ATG, anti-thymocyte globulin; EC-MPS, enteric-coated mycophenolate sodium; IL-2 RA, interleukin-2 receptor antagonist; PPH, plasmapheresis

Supplementary Table S2 Cyclosporine A trough levels for the study cohort

	IL-2 RA+CsA (N=29)
**Time point**	**CsA trough level [µg/mL] (mean±SD)**
**30d**	181 (±52)
**90d**	185 (±49)
**180d**	152 (±45)
**360d**	140 (±45)

CsA, Cyclosporine A; Il-2 RA, interleukin-2 receptor antagonist; N, number; SD, standard deviation

Supplementary Table S3 Correlation of torque teno virus load to cytomegalovirus and BK virus loads

**Variable**	**CMV/BKV positive samples**		
**Correlation***	**Analyzed Pairs (N)**	**Spearman's rho (95% CI)**	***P* value**
CMVL/TTVL	19	0.13 (-0.46–0.56)	0.6037 (ns)
BKVL/TTVL	55	0.38 (0.12–0.59)	0.0042 (**)

**Viral Loads were only correlated if available at the same time.*

BKV, BK virus; BKVL, BK virus load; CMV, cytomegalovirus; CMVL, cytomegalovirus load; N, number

Supplementary Figure S1 Infection and rejection risk stratified by induction therapy and torque teno virus load

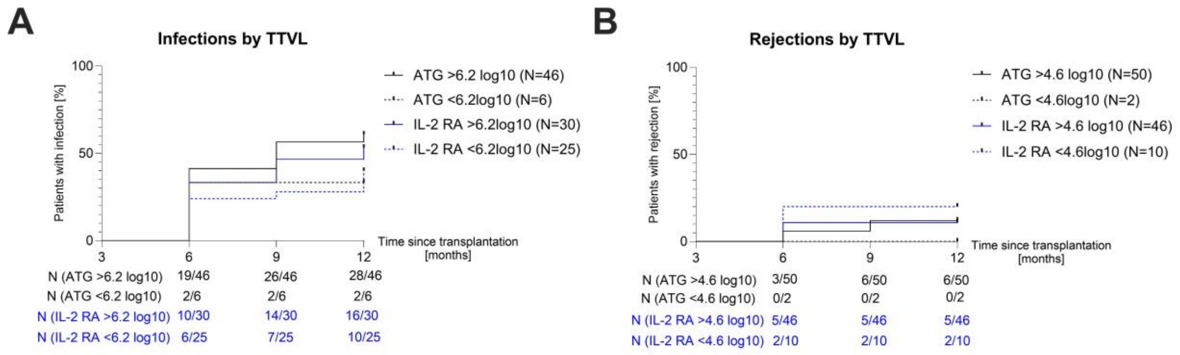

(A) Survival curves showing the incidence of hospitalization due to infection in the months 3–12 post-transplant in patients with ATG (black curves) and IL-2 RA (blue curves) induction therapy. Solid curves show patients with TTVL above the proposed upper limit (6.2 log_{10}) at 90 days post-transplant, while the dashed curves show patients with TTVL below the proposed upper limit (6.2 log_{10}) at 90 days post-transplant.

(B) Survival curves showing the incidence of rejection events in the months 4–12 post-transplant in patients with ATG (black curves) and IL-2 RA (blue curves) induction therapy. Solid curves show patients with TTVL above the proposed lower limit (4.6 log_{10}) at 90 days post-transplant, while the dashed curves show patients with TTVL below the proposed lower limit (4.6 log_{10}) at 90 days post-transplant.

Supplementary Figure S2 Infection and rejection risk according to 6-month torque teno virus load

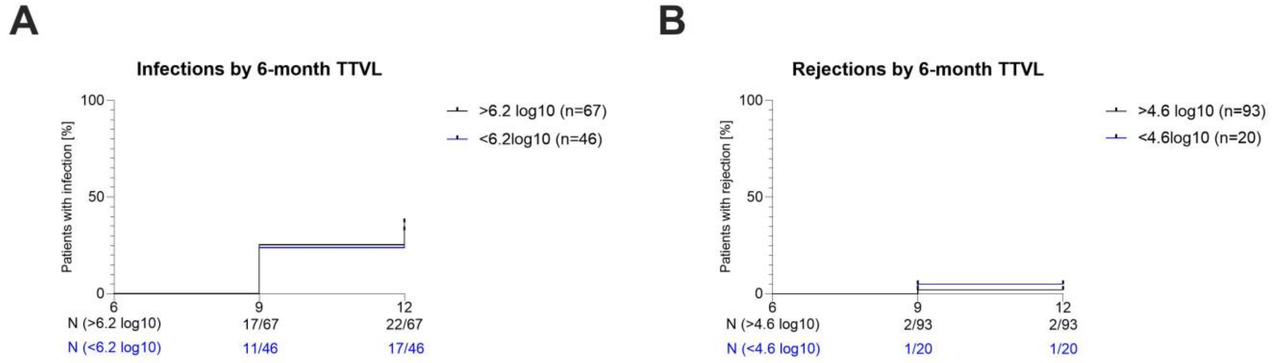

(A) Survival curves showing the incidence of hospitalization due to infection in the months 9–12 post-transplant in patients with TTVL above (black curve) and below (blue curve) the proposed upper limit (6.2 log_{10}) at 180 days post-transplant.

(B) Survival curves showing the incidence of rejection events in the months 9–12 post-transplant in patients with TTVL above (black curve) and below (blue curve) the proposed lower limit (4.6 log_{10}) at 90 days post-transplant.

Supplementary Figure S3 Infection sites and pathogens in the first year post-transplant

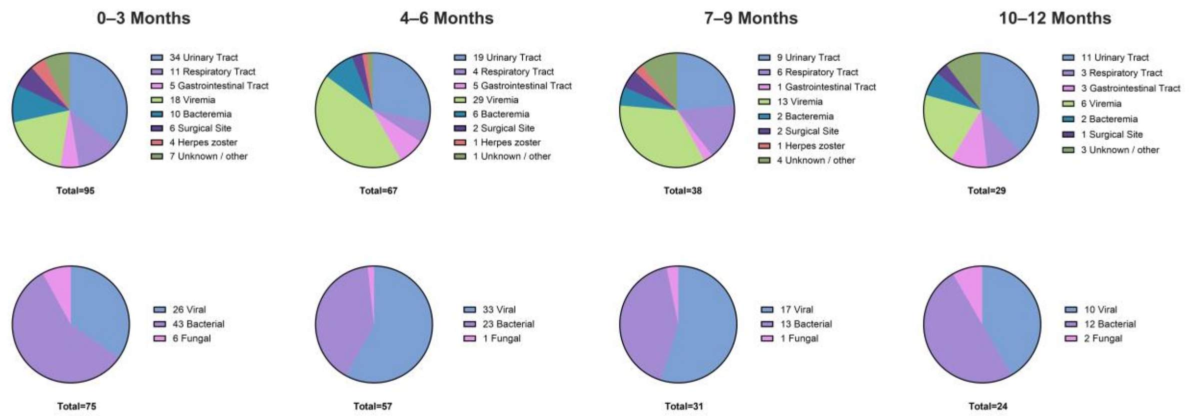

Infection sites and pathogens shown in three-month intervals (0–3 months, 4–6 months, 7–9 months, and 10–12 months post-transplant).

Supplementary Figure S4 Specific pathogens detected in the first year post-transplant

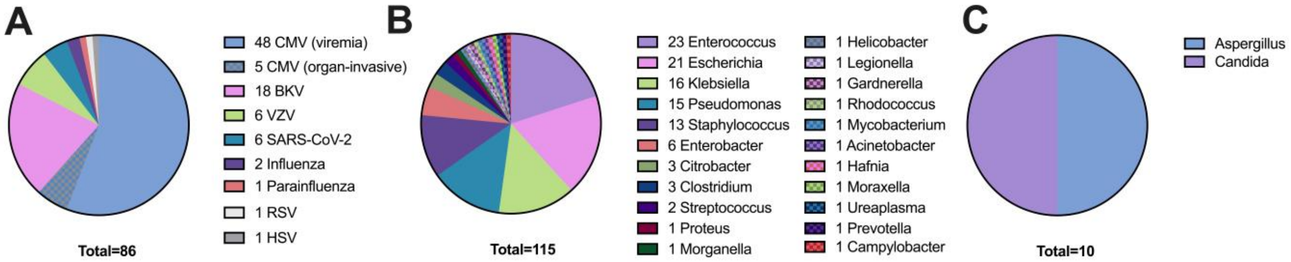

(A) Viral pathogens detected in the first year post-transplant.

(B) Bacterial pathogens detected in the first year post-transplant.

(C) Fungal pathogens detected in the first year post-transplant.
